# Research objectives and general considerations for pragmatic clinical trials of pain treatments: IMMPACT statement

**DOI:** 10.1097/j.pain.0000000000002888

**Published:** 2023-03-22

**Authors:** David J. Hohenschurz-Schmidt, Dan Cherkin, Andrew S.C. Rice, Robert H. Dworkin, Dennis C. Turk, Michael P. McDermott, Matthew J. Bair, Lynn L. DeBar, Robert R. Edwards, John T. Farrar, Robert D. Kerns, John D. Markman, Michael C. Rowbotham, Karen J. Sherman, Ajay D. Wasan, Penney Cowan, Paul Desjardins, McKenzie Ferguson, Roy Freeman, Jennifer S. Gewandter, Ian Gilron, Hanna Grol-Prokopczyk, Sharon H. Hertz, Smriti Iyengar, Cornelia Kamp, Barbara I. Karp, Bethea A. Kleykamp, John D. Loeser, Sean Mackey, Richard Malamut, Ewan McNicol, Kushang V. Patel, Friedhelm Sandbrink, Kenneth Schmader, Lee Simon, Deborah J. Steiner, Christin Veasley, Jan Vollert

**Affiliations:** a Pain Research, Department of Surgery & Cancer, Faculty of Medicine, Imperial College London, London, United Kingdom; b Department of Family Medicine, University of Washington and Kaiser Permanente Washington Health Research Institute, Seattle, WA, United States; c Department of Anesthesiology and Perioperative Medicine, University of Rochester Medical Center, Rochester, NY, United States; d Anesthesiology and Pain Medicine, University of Washington, Seattle, WA, United States; e Department of Biostatistics and Computational Biology, University of Rochester, Rochester, NY, United States; f VA Center for Health Information and Communication, Regenstrief Institute, and Indiana University School of Medicine, Indianapolis, IN, United States; g Kaiser Permanente Washington Health Research Institute, Seattle, WA, United States; h Harvard Medical School, Boston, MA, United States; i Department of Biostatistics, Epidemiology, and Informatics, University of Pennsylvania, Philadelphia, PA, United States; j Departments of Psychiatry, Neurology and Psychology, Yale University, New Haven, CT, United States; k Neuromedicine Pain Management and Translational Pain Research, University of Rochester School of Medicine and Dentistry, Rochester, NY, United States; l Department of Anesthesia, University of California San Francisco School of Medicine, San Francisco, CA, United States; m Kaiser Permanente Washington Health Research Institute and Department of Epidemiology, University of Washington, Seattle WA, United States; n Departments of Anesthesiology & Perioperative Medicine, and Psychiatry, University of Pittsburgh School of Medicine, Pittsburgh, PA, United States; o American Chronic Pain Association, Rocklin, CA, United States; p Department of Diagnostic Sciences, School of Dental Medicine, Rutgers University, Newark, NJ, United States; q Department of Pharmacy Practice, Southern Illinois University Edwardsville, Edwardsville, IL, United States; r Department of Neurology, Harvard Medical School, Boston, MA, United States; s Department of Anesthesiology and Perioperative, University of Rochester, Rochester, NY, United States; t Departments of Anesthesiology & Perioperative Medicine, Biomedical & Molecular Sciences, Centre for Neuroscience Studies, and School of Policy Studies, Queen's University, Kingston, ON, Canada; u Department of Sociology, University at Buffalo, State University of New York, Buffalo NY, United States; v Hertz and Fields Consulting, Inc, Silver Spring, MD, United States; w Eli Lilly and Company, Indianapolis, IN, United States; x Center for Health and Technology (CHeT), Clinical Materials Services Unit (CMSU), University of Rochester Medical Center, Rochester, NY, United States; y National Institutes of Health, Bethesda, MD, United States; z Departments of Neurological Surgery and Anesthesia and Pain Medicine, University of Washington, Seattle, WA, United States; aa Department of Anesthesiology, Perioperative, and Pain Medicine, Neurosciences and Neurology, Stanford University School of Medicine, Palo Alto, CA, United States; bb Collegium Pharmaceuticals, Stoughton, MA, United States; cc Department of Pharmacy Practice, Massachusetts College of Pharmacy and Health Sciences, Boston, MA, United States; dd Department of Anesthesiology and Pain Medicine, University of Washington, Seattle, WA, United States; ee Department of Neurology, Washington DC Veterans Affairs Medical Center, Washington, DC, United States; ff Department of Neurology, George Washington University, Washington, DC, United States; gg Department of Medicine-Geriatrics, Center for the Study of Aging, Duke University Medical Center, and Geriatrics Research Education and Clinical Center, Durham VA Medical Center, Durham, NC, United States; hh SDG, LLC, Cambridge, MA, United States; ii Chronic Pain Research Alliance, North Kingstown, RI, United States; jj Pain Research, Department of Surgery and Cancer, Imperial College London, London, United Kingdom; kk Division of Neurological Pain Research and Therapy, Department of Neurology, University Hospital of Schleswig-Holstein, Campus Kiel, Germany; ll Department of Anaesthesiology, Intensive Care and Pain Medicine, University Hospital Muenster, Muenster, Germany; mm Neurophysiology, Mannheim Center of Translational Neuroscience (MCTN), Medical Faculty Mannheim, Heidelberg University, Heidelberg, Germany

**Keywords:** Clinical trial, Clinical research methods, Pragmatic trials, Comparative effectiveness research, Pain, Analgesia

## Abstract

Many questions regarding the clinical management of people experiencing pain and related health policy decision-making may best be answered by pragmatic controlled trials. To generate clinically relevant and widely applicable findings, such trials aim to reproduce elements of routine clinical care or are embedded within clinical workflows. In contrast with traditional efficacy trials, pragmatic trials are intended to address a broader set of external validity questions critical for stakeholders (clinicians, healthcare leaders, policymakers, insurers, and patients) in considering the adoption and use of evidence-based treatments in daily clinical care. This article summarizes methodological considerations for pragmatic trials, mainly concerning methods of fundamental importance to the internal validity of trials. The relationship between these methods and common pragmatic trials methods and goals is considered, recognizing that the resulting trial designs are highly dependent on the specific research question under investigation. The basis of this statement was an Initiative on Methods, Measurement, and Pain Assessment in Clinical Trials (IMMPACT) systematic review of methods and a consensus meeting. The meeting was organized by the Analgesic, Anesthetic, and Addiction Clinical Trial Translations, Innovations, Opportunities, and Networks (ACTTION) public–private partnership. The consensus process was informed by expert presentations, panel and consensus discussions, and a preparatory systematic review. In the context of pragmatic trials of pain treatments, we present fundamental considerations for the planning phase of pragmatic trials, including the specification of trial objectives, the selection of adequate designs, and methods to enhance internal validity while maintaining the ability to answer pragmatic research questions.

## 1. Introduction

Pragmatic clinical trials are designed to answer research questions directly relevant to clinical or health policy decision-making.^46,117^ Examples include comparing the relative effectiveness of established treatment options under everyday clinical circumstances or answering research questions related to clinical processes, such as strategies of treatment delivery, dosing, interactions between interventions, or stepped-care approaches.

Pragmatic trials have become increasingly common in the field of pain research and other areas^53,62,81,118^ because the narrow remit of traditional placebo-controlled trials cannot answer the full range of clinical questions. For example, a pragmatic trial is valuable to assess whether a given therapy works as well as, or better than, established care when studied in a broad population and in nonacademic settings,^22^ regardless of the underlying mechanisms of benefit. Such research questions are most pertinent for therapies with an established efficacy and safety profile. They are also commonly formulated for therapies that have limited evidence of efficacy but are already widely used in clinical practice, and where the potential for harm is judged to be low, such as many complementary and integrative therapies.^53,62^ Particularly in chronic pain-related research, pragmatic trials may overcome limitations of trials with stringent eligibility criteria by better reflecting the realities of clinical practice, which often include patients with multiple comorbidities, high levels of disability,^3,35,76^ or socioeconomic barriers to treatment participation.^67,69,84^ Finally, pragmatic trials may provide more realistic effect size estimates and enhance translation of research findings into clinical practice.^92^ Key terms relevant for this article are defined in Box 1.

Box 1. **Glossary of relevant terms.**

**Table:** 

Term	Definition
Effectiveness	Effectiveness assesses whether an intervention is beneficial when provided under usual circumstances of healthcare practice (“Does it work in practice?”)^55^
Pragmatic RCT Used interchangeably with “effectiveness RCT”	An RCT intended to directly inform clinical or health policy decision-making, usually investigating therapeutic effectiveness or comparative effectiveness under conditions similar to clinical practice^46,82,117^
Real-world evidence	“Information on health care that is derived from multiple sources outside typical clinical research settings, including electronic health records […], […] billing data, […] registries, and […] through personal devices and health applications.” (p. 2293).^119^ Real-world data are distinct from pragmatic trial data, in that the latter are collected in a specifically designed research paradigm and real-world evidence from unmodified clinical practice.
Generalizability Used interchangeably with “external validity” and “applicability”^109,110^	The degree to which trial results may be considered valid in and applicable to participants, practitioners, interventions, outcome measures, and settings outside the respective trial^30,109,110^
Internal validity	“Internal validity describes the […] accuracy of the study results by minimizing error. Thus, internal validity is the degree to which changes in the dependent variable can be attributed to the intervention and is maximized by decreasing bias using design features such as random assignment, allocation concealment, and blinding” (p. 164)^140^
Efficacy	Efficacy is the extent to which an intervention provides benefit under ideal circumstances (“Can it work?”)^55^
Explanatory RCT Used interchangeably with “efficacy RCT”	An RCT that tests the benefits and/or harms of a treatment under relatively ideal conditions, aimed primarily at investigating a scientific or biological problem^46,82,117^
Mechanistic RCT	An RCT that investigates treatment mechanisms under relatively ideal conditions (alongside benefits and harms or exclusively)

RCT, randomized controlled trial.

While pragmatic trials are frequently portrayed as methodologically distinct from traditional explanatory randomized controlled trials (RCTs), a more suitable conceptualization is to view the role of RCTs on an explanatory-pragmatic spectrum.^82,98,127^ One end of the spectrum represents highly explanatory RCTs, which focus on answering mechanistic research questions and on evaluating efficacy and safety, often comparing treatments with placebo controls in a relatively homogeneous population. The other end of the spectrum represents RCTs with pragmatic aims.^82^ It is more helpful to examine the research question rather than individual trial methods to determine the pragmatism of trials because it is the research question which informs the choice of trial design and methods.^92^ In this sense, the distinction between pragmatic and explanatory depends on a trial's ability to answer a particular type of research question. Explanatory trials commonly ask efficacy questions (Box 1). This requires the trial design to control for effects of variables other than the studied intervention components, eg, by using placebo controls and narrow eligibility criteria.^61^ Pragmatic trials, because of their emphasis on enhancing the generalizability of findings, are frequently designed to reproduce routine clinical care^112^ or are embedded within it.^73^

In pragmatic trials, researchers have to achieve a balance between methods known to enhance internal validity and methods that align the trial with normal clinical practice. The appropriateness of “real-world” methods, such as flexible treatment delivery, depends on the question being asked and the intervention being tested. However, even if the research question is one of effectiveness, methods from normal clinical practice may unnecessarily compromise researchers' ability to interpret the findings. Mitigating steps may be possible that do not interfere with the trial's ability to answer a pragmatic research question. For example, while reflective of normal clinical practice, relatively flexible approaches to treatment delivery may mean that it is not clear whether participants actually received the allocated interventions and to what extent. In this case, one may conclude that the treatment is or is not effective. Only monitoring of protocol adherence, participant drop-out, or use of concomitant treatments would help determine whether these effects were due to the treatment or other confounding factors. “This information is not only relevant to interpret findings but also ‘pragmatic’ as it can inform implementation and intervention development”.

As Ford and Norrie noted in an influential 2016 article,^46^ “*Pragmatism should not be synonymous with a laissez-faire approach to trial conduct. The aim is to inform clinical practice, and that can be achieved only with high-quality trials*” (p. 462). Instead of dichotomizing into explanatory and pragmatic trials, these authors call for trials that adequately state and address their main objectives, including informing clinical practice. Therefore, each design choice requires consideration of at least 2 factors: its relation to the research question and its effects on trial quality.

This article presents considerations to help clinical pain researchers to optimize the balance between internal and external validity when they develop their trial design and methods (Box 2). Drawing on examples from pain research wherever possible, the article discusses fundamental considerations for the planning phase of pragmatic trials. These considerations include the clarification of trial objectives to facilitate the appropriate choice of design features, a summary of available trial designs, and several items relevant to increase a trial's internal validity, including available blinding and randomization methods. A second paper will discuss more specific research methods for conducting pragmatic trials of pain treatments. For example, this follow-up paper will include discussions of treatment delivery, comparator and control conditions, patient populations and study sites, outcome measures, study monitoring, and approaches to data analysis. Together, these publications will present best-practice research methods, proposing considerations for specific challenges and introducing methods to enhance the quality and value of pragmatic clinical trials.

Box 2. **List of core considerations for pragmatic trials in pain interventions. Individual points are elaborated on in the article text.**

**Table:** 

Clarify the objectives of the trial, including the appropriateness of a generally pragmatic vs a more explanatory approach
Ensure that design choices allow trial objectives to be met This includes • considering whether adaptive trial designs and other less commonly used designs may better answer the research question than traditional parallel-group designs, • using the PRECIS-2 tool^82^ and additional considerations presented here to evaluate design choices on the explanatory–pragmatic continuum during the planning phase of a trial, and • balancing of pragmatic design choices against more controlled approaches in the context of the research question
Report trial conduct and findings using the CONSORT extension for pragmatic trials^148^ and all other relevant extensions
Consider publishing the completed PRECIS-2 table^101^ along with justifications for design decisions in the context of the specific trial^92^

CONSORT, Consolidated Standards of Reporting Trials; PRECIS-2, Pragmatic–Explanatory Continuum Indicator Summary-2.

## 2. Methods of manuscript development

On October 22 and 23, 2020, a videoconference consensus meeting was held by the Initiative on Methods, Measurement, and Pain Assessment in Clinical Trials (IMMPACT), under the auspices of the Analgesic, Anesthetic, and Addiction Clinical Trials, Translations, Innovations, Opportunities, and Networks (ACTTION) public–private partnership with the U.S. Food and Drug Administration. Meeting participants were invited by the IMMPACT steering committee based on their expertise or experience involving pragmatic trials and to represent stakeholders from patient organizations, public institutions (such as the FDA and the National Institute of Health), and industry. In addition, all members of the ACTTION management, steering, executive, and oversight committees were invited. The meeting's objectives were to discuss important considerations and provide best-practice suggestions regarding the design, implementation, interpretation, and evaluation of pragmatic clinical trials of pain treatments to inform the planning, conduct, and reporting of such studies. Three consensus discussions were informed by nine 25-minute presentations by content experts and co-authors of this article. Presentations included the following topics: definitions and general considerations (L.D.), statistical approaches (S.E.), lessons learnt from pragmatic trials in various settings (A.W. and R.K.), study population definition and patient recruitment (J.M. and M.B.), study sites (J.F.), concomitant and rescue treatments (M.R.), and outcome domains and measures (M.B.). Furthermore, the results of a systematic review were presented (D.H.-S.).^62^ All participant details, lecture slides, and meeting transcripts are available on the IMMPACT web site, http://www.immpact.org/meetings/Immpact24/participants24.html. After the meeting, the first author drafted a consensus manuscript that was then reviewed by the co-authors. The reviewed materials and meeting discussions were then categorized into general and specific considerations with extensive internal manuscript reviews. The recommendations in this article are the product of vigorous discussions at the consensus meeting and continued iterative revisions of multiple draft manuscripts that were circulated among all the authors. The issues that required the most attention addressed the distinctions between pragmatic trials that are designed to meaningfully inform clinical practice and trials that prioritize the evaluation of treatment efficacy. The major concerns included the extent to which pragmatic trials can and should focus on bias control, including the measurement of expectations, as well as the relevance of clinical trial designs other than parallel-group RCTs and their congruency with pragmatic objectives.

## 3. Methods in current pragmatic trials of pain treatments

The background for these best-practice considerations is provided by a systematic review of methods of 57 self-labelled “pragmatic” or “comparative effectiveness trials” of pain treatments.^62^ Typically, such trials were multisite comparisons of 2 or more treatments, conducted across a broad spectrum of settings, recruiting several hundred participants living with chronic, mainly musculoskeletal pain and involving follow-up periods of 1 year on average. In the reviewed trials, complex nonpharmacological interventions were often studied, such as manual and physical therapies or acupuncture (28%) and cognitive-behavioral or other psychological interventions (16%). Twenty-one percent of trials investigated pharmacological treatments, 12% surgery, and a small percentage evaluated miscellaneous approaches such as multidisciplinary care, mind–body therapies, education, or alterations in general practice procedures. The most common comparators were another active intervention or “treatment as usual.” Participants were usually individually randomized, but 10% of trials used cluster randomization. Most trials were designed as superiority trials, aiming to detect a significant difference in outcomes between groups. Less than 10% were noninferiority or equivalence trials. Blinding of participants to group allocation was reported in a quarter of the trials (n = 13), with 3 studies “blinding” participants by randomizing trial practices and not requiring participant consent,^9,21,31^ others comparing 2 treatments that were indistinguishable to patients,^28,43^ or a “cohort multiple” design^104^ where patients were unaware of alternate study conditions.^139^ Seven of the reviewed trials reported single-blinding or double-blinding by means of placebo or attention control groups.^1,5,7,48,93,133,144^ Outcome assessments were almost always blinded.

To assess design features of the reviewed trials across the pragmatic–explanatory spectrum, the Pragmatic–Explanatory Continuum Indicator Summary (PRECIS)-2 tool was used. This tool considers 9 domains of trial design on a spectrum from very explanatory (scored as “1”) to very pragmatic (or similar to usual practice in the field; scored as “5”). The methodological domains assessed by PRECIS-2 include eligibility criteria, recruitment methods, trial settings, expertise and resources used to deliver interventions, flexibility of delivery and adherence, follow-up methods, primary outcome choice, and the method of primary analysis.^82^ Across the sample of 57 recently published trials of pain treatments, the average PRECIS-2 ratings per domain ranged from 3.0 (SD 1.6) for recruitment, indicating considerable effort to recruit participants, to 4.5 (SD 1.0) for outcomes, indicating that primary outcome measures were typically clinically relevant.^62^

Beyond characterizing recently published pragmatic trials of pain treatments, the review highlighted several areas for improvement in methodology and reporting, such as providing clear rationales about the choice of trial methods. As a major methodological challenge, trial feasibility and validity had to be balanced with attempts to interfere minimally with routine care. Researchers responded to this challenge in often creative ways or by sacrificing one aspect for the other, for example, using more elaborate recruitment methods at the expense of “pragmatism,” as defined by PRECIS-2, but ensuring successful recruitment or recruitment targeted to their research question. Relatedly, pragmatic design choices were prioritized differently or were harder to achieve in some PRECIS-2 domains than in others. Trial sites generally were judged to be better organized and equipped than what would be expected in usual practice and follow-up intensity often exceeded normal practice (also see 53). Challenges to trial pragmatism partly depended on the trial's specific circumstances, for example, with trials of drug therapies using more treatment standardization or chronic pain studies investing more efforts into patient recruitment. This systematic evaluation of current methods illustrates the balancing act faced by trial designers: to answer pragmatic research questions while exerting a sufficient level of control for successful trial completion and research validity.

## 4. Consensus statement of best-practice considerations

### 4.1. Clarifying trial objectives

When considering a pragmatic attitude to trial design, researchers ought to clarify the appropriateness of and motivation for a pragmatic trial, including an appraisal of available efficacy and mechanistic literature. With a clearly defined study intention, the most appropriate design choices can be made.^92^

Nonblinded comparative effectiveness trials provide different kinds of information than placebo-controlled efficacy RCTs. Because both kinds of information are important, before testing the effectiveness of new treatments in routine practice, existing efficacy and safety evidence for a treatment or for core components of a multimodal treatment ought to be considered to determine whether more efficacy research is needed. Although sufficient efficacy and safety data are required for new drug approval,^45^ trials comparing the effectiveness of existing nonpharmacological therapies are regularly conducted in the absence of high-quality efficacy research.^138^ Whether this is appropriate depends on the research question and trial context. For example, devising credible control groups is a major challenge for trials of nonpharmacological therapies, distinguishing treatment-specific effects of interest from other effects.^91,99^ Indeed, blinding difficulties have been used to justify unblinded comparative effectiveness designs.^15,138^ To overcome this challenge, specific guidance is becoming available for nonpharmacological trials^6,16^ most recently a comprehensive guideline by Hohenschurz-Schmidt et al.^63^ In addition, when low-risk treatments are already widely used, it is sometimes difficult to justify the need to evaluate against placebo. In these cases, comparing effectiveness with another commonly used modality can be considered. In other circumstances, a pragmatic (ie, relevant to clinical or policy decision-making) research question may require a sham-controlled trial (see 7; also discussed below).

The choices of appropriate study design and methods depend on the pragmatic research question and the corresponding testable hypothesis.^47^ There are several categories of comparative trials (see 80 for a definition of terms):

(1) Superiority of treatment A vs control group (eg, usual care or a specifically designed control condition).
(2) Superiority of treatment A vs treatment B.
(3) Noninferiority of treatment A vs treatment B.
(4) Equivalence of treatment A vs treatment B.

Additional specific objectives that could be considered pragmatic and assessed include

(1) assessing different treatment-delivery strategies (eg, stepped, stratified, or matched care)^50,73^;
(2) testing effectiveness in different care settings and populations;
(3) evaluating differential effects for patient subgroups, phenotypes, and other questions aimed at personalized care; and
(4) questions of risk-benefit, cost-effectiveness, and other clinically relevant composite outcomes.

Finally, pragmatic research goals are often informed by researcher engagement with key stakeholders (clinicians, healthcare leaders, and patients).

In summary, trial designers should clarify their research question considering existing evidence and current practice and assess and justify whether a pragmatic attitude to trial design is warranted.

### 4.2. Meeting trial objectives with high-quality designs

The main goal of a pragmatic approach to trial design is to answer a pragmatic research question^147^ in a scientifically robust manner, producing clinically impactful evidence. The overall trial design is thus guided by how trial results will be used. Study objectives should be achievable using the proposed trial methods, whether that means that the trial is closely aligned to typical clinical practice or not.

The precision of treatment effect estimates decreases with increasing trial heterogeneity, which is introduced, for example, by broad patient eligibility criteria, involvement of multiple trial centers, unregulated concomitant treatments, and flexible treatment application.^88,121^ Variability that reflects clinical practice is desirable in pragmatic trials but may pose a challenge to the interpretation of trial results, for example, understanding *why* an intervention was found to be (in)effective. Notably, this may be explained by how much different patient subgroups contribute to findings or other information relevant for clinical practice, such as low adherence to treatment protocols. There are several ways that such challenges of pragmatic trials may be turned into an advantage. For example, differential effects in specific subgroups (eg, age, sex, comorbidities, and concomitant treatments) can be determined by designing the trial to include sample sizes large enough to permit adequately powered subgroup analyses.^98^ Heterogeneity that is not required to answer the research question may have to be reduced, controlled, or measured to help interpret outcomes. For example, a question may ask about effectiveness in a real-world population with realistic intervention prescription scenarios (ie, with no or minimal adherence requirements). In this instance, it may be desirable to assess adherence, collect information about concomitant treatments, or measure changes in other behaviors to better understand trial outcomes.

In summary, researchers designing pragmatic trials need to ensure the reliability of results. This is important to distinguish effective from ineffective treatments, or treatments with differing levels of effectiveness, and must be balanced with the aim of producing research that is clinically meaningful, relevant, and applicable. In general, possible sources of heterogeneity in pragmatic trials should be explored and pertinent data measured and included in analyses. At a minimum, the most relevant clinical confounders (eg, comorbidities and concomitant treatments) should be considered.

### 4.3. Balancing pragmatic and explanatory qualities

For each pragmatic trial, there is an “optimal balance point between the poles of pragmatic and explanatory qualities.”^73,135^ Each design decision should be carefully evaluated on this spectrum, resulting in a robust framework to answer pragmatic research questions. At the consensus meeting, there was agreement that PRECIS-2 is a useful tool to inform the process of designing individual aspects of a pragmatic trial. Trials can benefit from considering both internal and external validity (or generalizability) for each PRECIS-2 domain. Researchers may emphasize generalizability when required by the research question but should preserve internal validity as much as possible, drawing on other available tools to evaluate internal validity (such as the Cochrane risk of bias tool).^82,123^ Allowing and measuring heterogeneity where necessary but reducing it where possible is important. Trials that aim to align themselves as much as possible with clinical practice typically show the following design features across PRECIS-2 domains:

(1) eligibility criteria aimed at including a broad and representative patient population, eg, not excluding participants with common comorbidities;
(2) participant recruitment that uses common means to engage with patients (eg, referrals or patient-driven contact seeking);
(3) settings and organizations that provide routine care;
(4) flexibility in treatment delivery and relatively low requirements for adherence;
(5) allowing most concomitant medications (and other cointerventions);
(6) choosing outcomes that are relevant to patients; and
(7) analyzing all participants as randomized (intention-to-treat [ITT]).

Other considerations in designing pragmatic trials include:

(1) use of real-world data (RWD) for eligibility criteria definition and recruitment;
(2) considering combined (eg, risk benefit) as well as responder and other subgroup analyses in addition to primary analyses;
(3) simplifying outcome choice, such as using measures with few scales as opposed to multiple-question disability questionnaires; and
(4) using real-world data collection tools, including consideration of wearables and mobile data sampling.

Apart from trial methods usually aimed at enhancing generalizability or answering pragmatic research questions, it is worth discussing how 2 common design features that enhance internal validity may apply to conducting high-quality pragmatic trials: randomization and blinding.

### 4.4. Applying methods for internal validity to pragmatic trials

#### 4.4.1. Randomization

**Figure 1.**
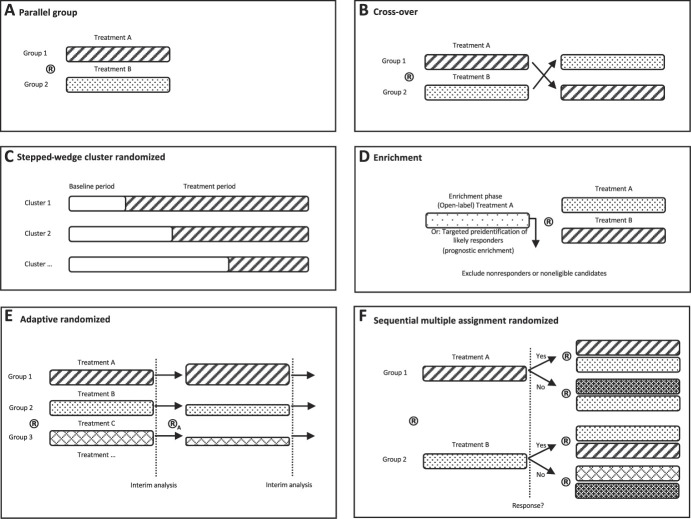
Schematic illustration of various group designs adaptable to pragmatic and comparative effectiveness trials. “Treatments” can also be other comparators or usual care, ® signifies randomization, and ®_A_ an adjusted randomization ratio. In panel (E), different box widths illustrate different group sizes after the randomization ratio has been adjusted in response to interim analysis.

Randomization is an essential design feature to enhance the probability that study groups are balanced in known and unknown factors that could affect treatment response. Related to randomization, allocation concealment may reduce bias,^25^ whereas stratification and blocking can increase precision, if applicable. Various randomization methods exist and may be considered to answer pragmatic questions:

(1) Cluster randomization involves randomizing entities or “clusters” other than individual patients—frequently trial centers, clinics, therapy providers, or geographic areas—and has been used in pragmatic pain trials.^62^ The choice of cluster depends on the level of intervention implementation, which may be easiest to perform and control at the clinic level. However, cluster randomization may be inappropriate when there is considerable variability in clinic size and characteristics. Another threat to validity is when the unit of allocation (cluster level) is different from the unit of outcome assessment (patients). When opting for cluster randomization, trialists need to be aware of possible selection bias arising when the assigned intervention is known during patient recruitment. To mitigate selection bias, baseline differences for potentially important predictors of treatment response should be assessed.^13^ Where possible, cluster randomized trials should recruit participants before site randomization to avoid selection bias. Irrespective of selection bias, trialists can recruit more clusters with fewer patients per cluster to enhance power.^112^
(2) Pragmatic research questions may invite researchers to consider other options to simple randomization. For example, patient preferences can be important predictors of treatment response and adherence, thus shaping clinical decision-making. Including patient preferences during randomization can be implemented in various ways but requires sophisticated controlling mechanisms and analyses.^17,78^
(3) More complex randomization processes, stepped-wedge designs, and enrichment methods are discussed below (Fig. 1).

#### 4.4.2. Blinding and accounting for participant expectancies

For many pragmatic research questions, it is accepted that the nonspecific (eg, contextual or placebo) effects form part of treatments' real-world effectiveness. From this perspective, blinding may not be appropriate. Furthermore, a clinical decision may be between multiple interventions with doubtful evidence of efficacy but with different risks for harm or healthcare cost. These interventions are often well-established and commonly used in clinical practice. In this situation, nonblinded comparative effectiveness trials can answer important questions while not negating the usefulness of improved efficacy research. Overall, we recommend blinding participants to group allocation where efficacy data are inconsistent and where compatible with trial objectives.

When participants cannot be blinded to group allocation, blinding to study hypotheses is often possible, for example, by not disclosing study objectives.^40,62^ To preempt the possibility of “resentful demoralization” or “compensatory rivalry” in the unblinded allocation to a trial condition perceived as less desirable,^4,29^ participants can be given limited information about the trial design—within ethical standards. Alternatively, patient preferences for all trial arms may be evaluated in a preparatory phase. Zelen or encouragement designs address this problem.^64,120^ Relatedly, participants' expectations of treatment benefit may be considered.^71^ In some scenarios, participant blinding may not be relevant, eg, when comparing patient outcomes in clinics randomized to a potentially improved form of care with clinics continuing to provide usual care. Christian et al.^24^ proposed a useful framework for making blinding-related decisions in pragmatic trials.

Where outcomes are collected by study staff, the blinding of outcome assessors to treatment groups is considered essential; this is also the case when patient-reported outcomes are used to reduce the risk of assessor bias. For the same reason, it is desirable that patients can enter patient-reported outcome measures directly into data capture systems, for example, electronically, reducing the potential for bias from study staff. However, benefits of electronic data capture may have to be weighed against its challenges, such as potentially lower response rates, data incompleteness, variable participant literacy or numeracy, technology access, and data privacy.^83,131,144^

When blinding is not possible, techniques for minimizing potential bias are available. These will be discussed below.

#### 4.4.3. Other bias minimization methods relevant for pragmatic trials

As in other clinical trials, pragmatic trialists need to consider possible sources of bias. Although randomization is commonly used to reduce bias in pragmatic trials, other bias-reduction methods such as treatment standardization and blinding study participants to treatment may conflict with pragmatic trial objectives. Examples of relevant threats to internal validity are listed in Table 1 together with recommendations on how to address these in pragmatic trials. As discussed earlier, the possible solutions for bias control in Table 1 need to be examined for potential conflicts with a trial's pragmatic objectives. In this case, they may not be suitable or their implications for the generalizability of findings should be declared. Additional considerations can be found in Katz et al.^72^

**Table 1:** Possible bias in clinical research and proposed considerations for methods to minimize bias in pragmatic trials of pain treatments.

Bias	Possible solutions (and explanation)
Recruitment bias Recruiting predominantly or failing to recruit certain subgroups of eligible participants	• Enhanced recruitment or targeted recruitment strategies In pragmatic trials, recruitment bias is a problem when the trial fails to represent the clinical target population. If the research question requires a relatively representative sample, more effort may be required to recruit diverse participants^2^ even if recruitment methods no longer reflect standard clinical practice. For example, a trial may not be conducted in the eventual target setting and thus not have access to “normal” recruitment pathways. Results may still generalize to the populations typically seen in such settings if representative participants are deliberately targeted for recruitment
Selection bias Selection of study participants skewed by factors such as participant characteristics (similar to recruitment bias but mainly driven by study staff)	• Cluster randomized trials: site randomization after participant recruitment • (Partial) blinding of recruiting study staff^24^ • Monitor baseline differences and/or control for important covariates in the analyses
Allocation bias Biased allocation of participants to study arms^59^	• Effective allocation concealment^123^
Assessor bias Knowledge of treatment allocation that influences outcome measurements	• Blinded outcome assessment^24^ • Use of objective outcome measures,^24^ eg, actigraphy^142^ • Use of disability and quality of life outcomes • Use multiple follow-up assessments Although outcomes in clinical practice are typically evaluated by providers, this is rarely necessary for pragmatic research questions. Therefore, bias control should be considered (see text)
Attrition bias Asymmetrical participant loss between study arms for nonrandom reasons^94^	• Assess risk during pilot phase • Monitor reasons for attrition • Include patient preference Although low adherence to treatments is common in clinical practice, it may undermine the interpretability of pragmatic trial results. This risk needs to be weighed against the relevance of using low-touch (“pragmatic”) strategies to increase adherence Methods to evaluate reasons for participant loss do not interfere with pragmatic research questions and should thus be implemented, especially if adherence is not promoted
Biased interpretation and reporting of results Reporting bias typically refers to the selective reporting of positive results. Apart from withholding negative results, alternative analyses can be performed,^52^ and results can be misinterpreted or misrepresented^14,37^.	• Evaluate overall internal validity • Preregister trial and follow protocol • Accurately report nonsignificant results in superiority trials (not claiming comparable effectiveness) • Discuss limits of generalizability and avoid overgeneralization of findings • Adhere to reporting guidelines^130^ Because of potentially greater heterogeneity, pragmatic trials may require more extensive reporting and more nuanced discussion than explanatory RCTs. This includes providing relevant contextual information The generalizability of trial results is usually an educated judgment,^149^ requiring knowledge of influential population characteristics and eligibility criteria.^32^ When discussing generalizability, trial authors should report relevant information to permit assessment of external validity,^51,66,148^ ensure that claims are supported by data, and discuss study limitations

Please note that the potential biases listed in the table also apply to more explanatory trials, but they may pose particular challenges in trials designed to inform clinical or policy decision-making. Potential biases are listed in the left-hand column and potential approaches to minimize each bias on the right. The proposed solutions need to be examined for potential conflicts with a trial's pragmatic objectives, in which case they may not be suitable or their implications for generalizability of findings must be clearly reported by the study authors.

RCT, randomized controlled trial.

For some pragmatic research objectives and end points measurement, precision and related aspects of internal validity are less of a concern, either because adequate measurement precision is self-evident or because it is not needed to support the study purpose. Examples include comparing 2 or more treatment approaches for costs of care, duration of adherence, or time to an objective medical event or change in treatment.

### 4.5. Considering alternative study designs for pragmatic research questions

So far, this article has discussed the importance of clarifying the appropriateness and intention of a pragmatic trial design and of carefully weighing methods that replicate normal clinical practice against internal validity. The following section proposes alternative study designs to the parallel-group RCT that are adaptable to the purposes of pragmatic trials.

Pragmatic trials of pain treatments are almost exclusively parallel-group designs.^62^ However, some pragmatic research questions may be usefully addressed with variations of parallel designs, such as enrichment or adaptive designs, or cross-over designs (Fig. 1). To date, these designs are not common practice in pragmatic trials and may on occasion conflict with routine clinical practice. Although these designs are not always suitable and have limitations, their potential for effectively answering research questions relevant to clinical decision-making is underestimated. When planning a pragmatic trial, we suggest considering such alternative options for their potential to answer specific research questions and particularly whether they may increase efficiency and trial feasibility. Below, potential opportunities and limitations of alternative designs are presented. Further generic and pain-specific methodological guidance is available.^38,47^

#### 4.5.1. Large, simple trials

Large, simple trials (LSTs) are defined by large sample sizes, broad eligibility criteria, minimal data collection requirements, and use of objective, often routinely collected outcome measures. Large, simple trials are parallel-group trials believed most suitable for pharmacological postapproval effectiveness and safety research. Large, simple trials typically interfere little with clinical practice and maintain scientific rigor by randomizing participants to treatments with clinical equipoise.^105,107^ Their large size of often several thousand participants can provide high-quality information even for rare outcomes. Their minimal interference with routine care makes them broadly aligned with a “pragmatic” attitude to trial design. There are organizational challenges and large costs associated with establishing research networks large enough to support LSTs,^41,105,111^ although, once established, trials become much more cost efficient. Their reliance on objectively measurable end points, such as death or hospitalization, has likely hindered their implementation in pain research. Exceptions exist, which also illustrate the usefulness of integrated healthcare systems and electronic health records to facilitate clinical research.^21^ Especially regarding analgesic safety studies, the lack of LSTs is a missed opportunity. Given the prevalence of comorbidities and polypharmacy in people with persistent pain, LSTs might also provide valuable insights into drug interactions while avoiding some biases of observational studies. Potentially, improvements in electronic health records and simple mobile data collection methods will facilitate the broader adoption of LSTs in pain research.^11,111^

#### 4.5.2. Cross-over design

Provided certain assumptions regarding study treatments and medical conditions are met,^38^ cross-over designs may be useful for addressing pragmatic research questions. If components or application sequences within a complex treatment are to be assessed (eg, symptom-guided vs generalized manual therapy^42^), cross-over designs seem possible and may remove between-patient variance, reducing sample size requirements.^137^ Cross-over designs are rare in pragmatic pain trials^62^ and investigators typically choose parallel-group designs for pragmatic questions.^42,54,86,133^ For short-acting, non–disease-modifying drugs, cross-over designs may be used to answer pragmatic questions, respecting the usual methodological standards.^137^ Finally, switching between treatments after a certain time or on treatment failure is a common scenario in clinical practice and its effects can be assessed in pragmatic trials.^106,108^ Either the treatment sequence is randomized as in cross-over designs or a postrandomization treatment switch is triggered by clinical factors (not considered a traditional cross-over trial). The cross-over may act as an incentive during recruitment, mitigate patient disappointment in unblinded trials, or, under some circumstances, enable subgroup analyses. Individual (n-of-1) or multiperiod cross-over designs are adaptations geared towards clinical decision-making.^85,146^

#### 4.5.3. Stepped-wedge cluster randomized design

A variant of a cluster randomized trial is a stepped-wedge cluster randomized trial. Stepped-wedge cluster randomized trials are a pragmatic attempt to reconcile various stakeholders' needs and the practical constraints of large-scale intervention or policy implementation. Starting with a nonexposure period for all study clusters, this design involves “*random and sequential crossover of clusters from control to intervention until all clusters are exposed.*”^57^ Stepped-wedge designs have been successfully applied to study healthcare interventions during routine implementation of new approaches over time,^87^ including multimodal workplace interventions for low back pain^103^ and digital health psychological interventions for children and adolescents with chronic pain.^96^ Recent trials also studied effects of modifications in diagnostic procedures on healthcare utilization.^68^ With every cluster eventually exposed, the phased baseline period acts as the internal control condition and all clusters contribute to both study conditions—a notable advantage over traditional cluster randomization.

Stepped-wedge cluster randomized trials face challenges to reconcile practical constraints (eg, the speed or extent with which an intervention can be implemented and the transition periods that may arise) and methodological requirements (eg, sample size calculation, recruitment, concealment, potential dependence within clusters, calendar time effects, and repeated measures). Social and healthcare trends outside the trial may also affect interpretability as individual clusters are affected differently. Stepped-wedge trials provide more statistical power than parallel cluster designs when clusters are heterogeneous and/or large. Additional methodological guidance is available.^57^

#### 4.5.4. Enrichment designs

“Enrichment” refers to randomizing only patients with an increased likelihood of treatment success (practical, prognostic, or predictive enrichment^125^) or other specific characteristics. Targeted preidentification during eligibility screening, including the use of biomarkers, clinical diagnostics, and enrichment phases can be pragmatic if these methods address a clinically relevant question and are feasible in routine clinical practice. Examples of prognostic enrichment are trials of patients at high risk of developing chronic low back pain, as identified by a routinely available risk-stratification strategy such as the STarT-Back screening tool.^33,60^ However, although this process allows for pragmatic research questions, it certainly reduces generalizability by excluding a certain number of potential treatment recipients.^82^ Readers are referred to existing guidance for detailed discussions of enrichment strategies.^90,130^

#### 4.5.5. Adaptive and other designs responsive to accumulating trial information

Characterized by “*using results accumulating in the trial to modify the trial's course in accordance with prespecified rules,*”^97^ adaptive designs enable researchers to respond to interim safety and efficacy data. For example, if problems are encountered early in the trial, treatment intensity (eg, dose or number of treatment sessions) can be altered, randomization ratios changed, or treatment arms added or dropped, arguably saving research resources.^38^ To be feasible, effectiveness and safety outcomes must be expected to occur relatively early in the treatment course. Adaptive trials are challenging to conduct, both logistically and methodologically, and require expert biostatistician support. Regarding trial designs involving outcome (or response)-adaptive randomization, there are important limitations including bias and loss of efficiency of treatment effect estimators, bias caused by temporal trends in participant characteristics, volatility in sample size distributions with more participants assigned to the inferior treatment, potentially large imbalances in participant characteristics, greater potential for unblinding, and ethical concerns.^8,19,20,34,58,77,102,116,126^ Further theoretical and practical considerations of adaptive designs are available.^26,27^ Use of such designs must be accompanied by careful consideration of these limitations.

More relevant for pragmatic trials are designs of personalized or stepped-care approaches, or adding or dropping of study arms without losing the integrity of randomization.^115,136^ For example, the STAR*D (Sequenced Treatment Alternatives to Relieve Depression) trial^65,113,114^ tested subsequent treatments for nonresponders. The design was a precursor to Sequential Multiple Assignment Randomized Trial (SMART) designs,^79^ which mitigate some of the above concerns regarding adaptive randomization. Sequential Multiple Assignment Randomized Trial designs can be conceptualized as sequences of empirical trials of different interventions, often mimicking and thus informing clinical practice. STAR*D was conducted in 41 outpatient settings and enrolled over 4000 participants. The trial included 4 levels of randomization for patients who did not remit with a first course of citalopram for major depressive disorder, resulting in up to 4 treatment levels of various medications, switch and augmentation options, and cognitive therapy. Albeit a trial of depression, this design may be applied to stepped care and treatment alternatives for pain, such as those outlined in guidelines for painful conditions but rarely tested against one another (eg, commencing treatment with education, reassurance, and over-the-counter analgesics before considering physiotherapy, manual therapy, multimodal rehabilitation, etc^10^). In STAR*D, the numerous treatment options at level 2, and inclusion of treatment preferences (patients could choose a range of potentially assigned treatments), resulted in small group sizes, making comparisons difficult, and the absence of no-treatment controls should be noted. Conversely, including patient—and possibly provider—choice of treatment^12^ arguably reflects routine practice,^128^ as in a pragmatic trial of multiple or multimodal pain therapies.^39,124,129^ Integrating treatment choice into randomization algorithms has also been proposed as so-called equipoise-stratified randomization.^78^

Although not as elaborate as STAR*D, several ongoing pain trials use SMART designs to study clinically highly relevant issues, mainly related to tailoring of nonpharmacological pain management.^36,44,49,74,122^ For example, a trial of breast cancer compares different doses of a pain-coping skills program and dose adaptations depending on an initial (non-)response.^74^ However, this trial is designed to have adequate power only for the first treatment period and related analyses (ie, before rerandomization), underlining the logistical challenges of such designs. Studies that have adequate power for subsequent analyses after switching treatments are the ongoing OPTIMIZE trial, comparing physical therapy and cognitive behavioral therapy and recruiting 945 participants,^122^ and the SMART LBP study aiming for 1200 participants.^49^ Participant loss and nonadherence are major threats to all types of trials. In SMART designs, this problem may be heightened because of repeated randomization steps and consequently smaller groups. Published protocols of SMART designs thus document increased efforts and low PRECIS-2 ratings in the “adherence” domain (ie, treatment adherence is encouraged beyond normal clinical practice). Similarly, participants are followed up closely and recruitment is more elaborate.^49,122^ Finally, the “Determinants of the Optimal Dose and Sequence of Functional Restoration and Integrative Therapies study” investigates standard rehabilitation and complementary therapy approaches in a military setting.^44^ Most of these studies also aim to identify predictors of initial treatment responses.

### 4.6. Exemplary trials balancing internal and external validity

We have suggested strategies that do not always reflect current practice in the field.^62^ Primarily, we are calling for more attention to the balance between real-world applicability and internal trial validity. To illustrate how this can be performed effectively, we discuss 2 well-designed studies:

Beard et al.^7^ studied arthroscopic subacromial decompression for subacromial shoulder pain, using 30 sites and 38 operating surgeons of the UK National Health Service. When the trial was planned, there was insufficient evidence from efficacy trials. Nonetheless, shoulder arthroscopies were routinely performed in clinical practice, making real-world effectiveness a pertinent research question. To answer this question while safeguarding against expectancy-related effects, the trial included both a sham control group and a no treatment group. The control intervention enabled the distinction between placebo effects and normal disease course. The trial showed no difference between arthroscopy and sham but a clear benefit of both over no treatment. Further illustrating challenges of pragmatic trials and potential solutions, Beard et al.^7^ reported that they struggled with participants not receiving their allocated intervention. The clinical context and possibly patient preferences may explain these problems. For example, shoulder surgery patients may change their mind or surgery slots may not become available during the trial period. Had the researchers not included preplanned sensitivity analyses to assess the effects of intervention adherence, interpretation of findings would have become nearly impossible. Recently, Kerns et al.^75^ have advocated for investigators to find the right “balance” between the flexibility in treatment delivery and adherence monitoring that is consistent with clinical practice and the importance of building confidence in the fidelity of the independent variable, namely, the interventions being studied. The arthroscopy trial by Beard et al.^7^ addressed clinically relevant questions in a typical clinical environment, while using multiple features that are commonly considered priorities in explanatory trials (eg, blinding and per-protocol analyses^24,82^), which added valuable information. Balancing explanatory and pragmatic methods, the study reliably informs clinicians and policy decision-makers about the utility of a pain treatment in a realistic context. Albeit a single trial, this well-conducted study resulted in the change of clinical recommendations.^132^

Comparisons between 2 active treatments rather than with sham comparators are more typical for pragmatic pain trials.^62^ For example, Cherkin et al.^23^ compared mindfulness-based stress reduction (MBSR) to cognitive-behavioral therapy (CBT) and to usual care for patients with chronic low back pain. This trial balanced the considerations between internal and external validity well. With an overall PRECIS-2 rating of 3.3 (placing the overall design centrally between explanatory and pragmatic poles), the researchers prioritized clinically relevant outcome measures, pragmatic data analysis (intention-to-treat), and low study questionnaire burden. The trial used more explanatory methods in the domain “flexibility of intervention delivery,” ensuring with pretrial training of providers and continuous monitoring that the interventions were delivered according to the protocol (also see 75). This design feature was mainly driven by funding requirements. Furthermore, the trial used targeted participant recruitment and dedicated trial centers. In an otherwise “pragmatic” trial, this illustrates reasons for design decisions that deviate from usual care: the reduction of bias and practical constraints. In addition, with more control over intervention content, Cherkin et al. were able to draw more definite conclusions than with MBSR practitioners who all followed their own treatment preferences. Conversely, the trial's treatment protocol was later used for a university training program in MBSR, providing a nice example of research and clinical practice informing one another. Having aimed for a large study sample and experiencing recruitment and adherence difficulties, the practical requirement to complete the trial meant that recruitment methods typical for clinical practice needed to be bolstered. With a typical, moderate attendance of MBSR and CBT, the trial showed a benefit of these interventions over usual care. As the authors acknowledge, however, the absence of a sham or attention control group prevented the assessment of effect mediators. For example, such a control intervention could have elucidated the effects of specific intervention features or of the additional attention received from healthcare providers in the treatment group.

In summary, these studies illustrate how trials can be designed to answer clinically relevant questions in a rigorous manner. In addition, they illustrate the practical challenges and research constraints that can lead to methodological compromise. To reduce research waste through small, flawed, and thus uninformative trials, funding bodies should facilitate best-practice solutions.^18,41,141,143^

## 5. Discussion

Pragmatic trials of pain treatments are conducted to inform clinical decision-making and health policy for people living with pain. They address important clinical or policy questions about both pharmacological and nonpharmacological therapies. Because of large funding initiatives in the United States,^56,95,100^ pragmatic trials are likely to continue to gain in importance in the future. It remains a priority to find safer, more effective, and practical approaches to pain management and to advance personalized medicine. This article has outlined the consensus of a group of participants with expertise in the design, conduct, analysis, and/or interpretation of clinical trials. The fundamental design and methodological considerations for pragmatic trials emphasize the importance of balancing relevance for clinical practice (external validity) with ensuring scientific integrity (internal validity) of the trial results. Based on a systematic review of current research practice and in-depth discussions, we identify opportunities for improving the conduct of pragmatic trials, provide guidance on their design, and presented considerations for future trials. The basic notion is that measurable variables that account for heterogeneity should be identified and controlled or included in statistical modeling where the research question permits it; but heterogeneity should be accepted and incorporated into the trial design where required by the objectives of a trial. Study designs such as sequential multiple assignment or even cross-over designs are essentially absent from current pragmatic trials of pain therapies,^62^ despite their potential to inform clinical and policy decision-making.

This article is limited in that it has only presented general considerations and guidance. Trial researchers will have to consider each aspect of research designs and methods individually and in the context of their specific pragmatic research question and potential study setting. We have highlighted methods for minimizing bias in pragmatic trials while recognizing that choice of methods needs to consider their impact on generalizability of findings. More rigor in this regard will increase the value of pragmatic clinical trials in shaping clinical decision-making and health policy. Furthermore, the present considerations were not developed by formal consensus methodology,^89^ albeit being informed by a systematic review of current practice in pragmatic trials. In addition, not all individuals involved had expertise in pragmatic trials but all represented stakeholders, such as academics, industry, regulators, and patient initiatives, that have substantial investment in evaluating the effects and safety of pain treatments.

To date, the main guidance documents for pragmatic trials are the PRECIS-2 tool for the design^82^ and the CONSORT extension for the reporting of pragmatic trials.^148^ Another useful resource is the NIH Collaboratory's “Living Textbook” (https://rethinkingclinicaltrials.org/). For reporting, we suggest the CONSORT reporting guidance (and all other relevant CONSORT extensions) and believe that better adherence will increase the usefulness of pragmatic trials. For design considerations, however, the PRECIS-2 tool requires more nuanced discussion. The tool is certainly useful in helping guide the design of individual trials^70^ and we recommend its use, but researchers need to be aware that a high rating may not always be desirable for each domain. High ratings are given when the trial feature is comparable to routine clinical practice and lower ratings represent departures from the normal clinical procedures or scenarios. As our considerations emphasize, pragmatic trials attempt to answer pragmatic research questions by testing hypotheses about treatment effectiveness and do not necessarily closely reproduce clinical practice. For example, pain trialists may opt for real-world resemblance more in some domains than in others, often choosing more intensive recruitment methods to obtain a patient sample representative of the population of interest or performing more in-depth outcome assessments.^62^ Importantly, enhanced recruitment efforts may also be required for more representative or diverse samples. Finally, we strongly recommend that authors report their reasons for all such choices. Publishing the PRECIS-2 table (rather than the more commonly reported wheel diagram) is a good basis for such reporting^82,101^ and such information will be of value to readers and future trial designers.^62^

## Conflict of interest statement

The first author was renumerated by IMMPACT for their work at the consensus meeting and in drafting the manuscript. The project was supported by ACTTION, a public–private partnership. The views expressed in this article are those of the authors and no official endorsement by the Food and Drug Administration (FDA) or the pharmaceutical and device companies that provided unrestricted grants to support the activities of the ACTTION public–private partnership should be inferred. Individual authors' declarations of potential conflicts of interest are as follows: M. J. Bair reports grants or contracts from VA Health Services Research and Development, VA Cooperative Studies Program, and National Endowment for the Arts and participation on a Data Safety Monitoring Board or Advisory Board on a NIH project conducted at the University of Utah. This trial is a pragmatic trial of physical therapy intervention. Prof Cherkin reports being paid an honorarium for mentoring the first author with manuscript writing. L. L. DeBar reports support for the present manuscript from Kaiser Permanente Washington Health Research Institute (KPWHRI); grants and contracts from National Institutes of Health (NIH) and Patient-Centered Outcomes Research Institute (PCORI); an honorarium for a lecture at 2020 IMMPACT Consensus Meeting; support for attending meetings from KPWHRI and NIH; and participation on a Data Safety Monitoring Board or Advisory Board for NCCIH, BACPAC DSMB. P. Cowan is the co-founder and secretary of the World Patients Alliance and Board Member Emeritus as well as Founder of the American Chronic Pain Association. P. Desjardins reports grants or contracts from the NIH/NIDCR Opioid Analgesic Reduction Study as co-investigator; consulting fees from Acadia Pharmaceuticals, GlaxoSmithKline Consumer Healthcare, Neurana Pharmaceuticals, Bayer Consumer Healthcare, Senju USA, Antibe Therapeutics, and CenExel; payment or honoraria for lectures, presentations, speakers bureaus, manuscript writing, or educational events from Bayer Consumer Healthcare; participation on the GSK Consumer Advisory Board; and roles on the Board of Directors of ProSelect/Coverys Medical Liability Insurance and the Board of Governors of South Orange Performing Arts Center. R. H. Dworkin has received in the past 5 years research grants and contracts from the U.S. Food and Drug Administration and the U.S. National Institutes of Health, and compensation for serving on advisory boards or consulting on clinical trial methods from Abide, Acadia, Adynxx, Analgesic Solutions, Aptinyx, Aquinox, Asahi Kasei, Astellas, Beckley, Biogen, Biohaven, Biosplice, Boston Scientific, Braeburn, Cardialen, Celgene, Centrexion, Chiesi, Chromocell, Clexio, Collegium, CombiGene, Concert, Confo, Decibel, Editas, Eli Lilly, Endo, Ethismos (equity), Eupraxia, Exicure, GlaxoSmithKline, Glenmark, Gloriana, Grace, Hope, Hospital for Special Surgery, Lotus, Mainstay, Merck, Mind Medicine (also equity), Neumentum, Neurana, NeuroBo, Novaremed, Novartis, OCT, Orion, OliPass, Pfizer, Q-State, Reckitt Benckiser, Regenacy (also equity), Sangamo, Sanifit, Scilex, Semnur, SIMR Biotech, Sinfonia, SK Biopharmaceuticals, Sollis, SPRIM, Teva, Theranexus, Toray, Vertex, Vizuri, and WCG. R. R. Ewards reports no conflicts of interest. J. T Farrar reports grants or contracts from the NIH-NCATS-UL1 Grant (Co-I), FDA-BAA Contract, NIH-NIDDK-U01 Grant (CoI), and NIH-NINDS-U24 Grant (PI); consulting fees from Lilly and Vertex Pharma; participation on a Data Safety Monitoring Board or Advisory Board for NIH-NIA(DSMB); and a role as President-Elect US-ASP. M. Ferguson reports grants or contracts from ACTTION paid to her institution for work on systematic reviews and payment or honoraria for lectures at IMMPACT meetings from ACTTION. R. Freeman reports consulting fees from AlgoRx, Allergan, Applied Therapeutics, Clexio, Collegium, Cutaneous NeuroDiagnostics, Glenmark, GW Pharma, GlaxoSmithKline, Eli Lilly, Lundbeck, Maxona, Novartis, NeuroBo, Regenacy, Vertex, and Worwag and stock options in Cutaneous Neurodiagnostic Life Sciences, NeuroBo, Maxona, and Regenacy. J. S. Gewandter reports grants or contracts from the NIH; consulting fees from AlgoTX, GW Pharma, Magnolia Neurosciences, Orthogonal, Science Branding Consulting, AKP Pharma, and Eikonizo and support for attending meetings or travel from SOPATE and INS. I. Gilron declares a travel stipend to attend ACTTION meeting 2019 and reports consulting fees from CombiGene, GW Research, Lilly, and Novaremed. H. Grol-Prokopczyk reports grants or contracts from the National Institute on Aging of the National Institutes of Health, Award #R01AG065351; honoraria for an invited lecture for Multidisciplinary Research in Gerontology Colloquium Series, University of Southern California, and for an invited lecture at Napa Pain Conference; and travel reimbursement for travel to Napa Pain Conference. S. H. Hertz declares consulting fees from Adial Pharmaceuticals, AIXThera Operations Limited, Asahi Kasei Pharma America Corporation, Allay Pharmaceuticals, Amygdala Neurosciences, Araim Pharmaceuticals, Artugen Therapeutics, Avenue Therapeutics, BioQ Pharma, Cali Pharmaceuticals, Celero Pharma, Centrexion Therapeutics, Collegium Pharmaceutical, Concentric Analgesics, Currax Pharmaceuticals, Delpor, Domain Therapeutics, Enalare Therapeutics, Go Medical Industries, Heron Pharmaceuticals, Innocoll Biotherapeutics, Intra-Cellular Therapies, Kaleo, Lyndra Therapeutics, Maxona Pharmaceuticals, MDI Pharma, Nema Research, Neuroderm, Neumentum, Novilla Pharmaceuticals, OncoZenge, Pfizer, PleoPharma, SafeRx Pharmaceuticals, The Scripps Research Institute, Sollis Therapeutics, Sparian Biosciences, Teikoku Pharma USA, Taiwan Liposome Company, Tremeau Pharmaceuticals, Vallon Pharmaceuticals, Vizuri Health Sciences, and WinSanTor. D. G. Hohenschurz-Schmidt reports support for the present manuscript from a PhD studentship by the Alan and Sheila Diamond Charitable Trust and a honorarium from IMMPACT; a research grant from The osteopathic Foundation (paid to institution); consulting fees from Altern Health Ltd; and the role of executive committee member of the Society for Back Pain Research. S. Iyengar reports employment and travel support from the NINDS/NIH; stock options in Retiree and Eli Lilly and Company; and other financial or nonfinancial interests through employee of NINDS/NIH and adjunct senior research professor, Indiana University School of Medicine, Departments of Anesthesia and Clinical Pharmacology. C. Kamp reports support for the present manuscript from ACTTION, FDA contract #HHSF223201000078C, with payments made directly to her institution, University of Rochester Medical Center, providing 5% salary support and consulting fees from Clintrex Research Corporation (payments made to her consulting company CLKamp Consulting LLC with none of the consulting in relation to the indication of pain). B. I. Karp declares no conflict of interest. R. D. Kerns reports honoraria for presentation at the IMMPACT consensus meeting that informed this manuscript; research grants from NIH, PCORI, and VA paid to his institution; a consulting fee for a NIH-sponsored research grant; a honorarium for planning and participation in an IMMPACT consensus conference on patient engagement in clinical pain research; honoraria for participation in NIH and PCORI DSMBs and a honorarium for participation as member of Scientific Advisory Board, Chronic Pain Centre of Excellence for Canadian Veterans; an unpaid role on the Board of Directors, A Place to Nourish your Health; and an honorarium for role as the Executive Editor, *Pain Medicine*. B. A. Kleykamp reports income from ACTTION as a full-time employee October 2018-August 2021; being the owner and principal of BAK and Associates, LLC a research and science writing consulting firm; contracts to BAK and Associates, LLC over the past 36 months include STATinMED, American Society of Addiction Medicine, ECRI, Hayes/Symplr, Pinney Associates, and Palladian Associates; payments and honoraria for lectures, presentations, speakers bureaus, manuscript writing, or educational events from University of Kentucky, STATinMED, Filter magazine, and Virginia Commonwealth University; support for attending meetings or travel from University of Kentucky and Virginia Commonwealth University; and a role as Communications Chair for the College on Problems of Drug Dependence. J. D. Loeser reports no conflict of interest. S. Mackey reports support for the present manuscript from the National Institutes of Health, U.S. Food and Drug Administration, Patient-Centered Outcomes Research Institute, Chris Redlich Professorship in Pain Research, and Dodie and John Rosekrans Pain Research Endowment Fund (all through Stanford University); consulting fees from Oklahoma University-Smith NIH Grant; payments or honoraria for lectures, presentations, speakers bureaus, manuscript writing, or educational events from Memorial Sloan Kettering Cancer Center, National Institutes of Health, Washington University, Oakstone Publishing, Comprehensive Review of Pain Medicine CME Lecture Series, Walter Reed AFB, Web-Based Lecture, Bull Publishing, George Washington University, University of Washington, Veterans Affairs, HSRD Naloxone Distribution IIR Advisory Board, Canadian Pain Society, National Institutes of Health, and New York University; support for attending meetings or travel from American Academy of Pain Medicine, American Society of Regional Anesthesia and Pain Medicine, Washington University, George Washington University, National Institutes of Health, University of Washington, U.S. Federal Drug Administration, New York University, Weill Cornell Medical College, and the International Neuromodulation Society (INS); roles on the Drug Safety and Risk Management Advisory Committee, Anesthetic and Analgesic Drug Products Advisory Committee (DSaRM/AADPAC)/(FDA) (unpaid role as Advisory Committee Member) and for HSRD Naloxone Distribution IIR Veterans Affairs (VA) (honorarium paid to himself for a role as Advisory Board Member); an unpaid role as Vice-Chair Committee on Temporomandibular Disorders for the National Academies of Sciences, National Institutes of Health, (NAS)/(NIH); other financial interests through the National Institutes of Health for T32 Postdoctoral Fellows who conduct research in laboratory; and salary supported by NIH and administered through Stanford University. R. Malamut reports no conflicts of interest. J. D. Markman declares consulting fees from Lateral Pharma, Editas, Clexio Pharma, Nektar, Pfizer, Eliem, Biogen, and Lilly; participation on Data Safety Monitoring or Advisory Boards for Regenacy Pharmaceuticals, Tonix Pharmaceuticals, and Novartis Pharmaceuticals; roles as an ex officio board member of the North American Neuromodulation Society and Treasurer of the Neuromodulation SIG of IASP; and stock options in Yellowblack Corp and Flowonix Corp. M. P. McDermott declares grants or contracts from the NIH, U.S. Food and Drug Administration, Cure SMA, and PTC Therapeutics; consulting fees from Fulcrum Therapeutics, Inc, and Neuroderm, Ltd; and participation on a Data and Safety Monitoring Board or Advisory Board for the NIH, Eli Lilly and Company, Catabasis Pharmaceuticals, Inc, Vaccinex, Inc, Neurocrine Biosciences, Inc, Voyager Therapeutics, Prilenia Therapeutics Development, Ltd, ReveraGen BioPharma, Inc, and NS Pharma, Inc. E. McNicol reports grants or contracts from ACTTION paid to his institution for work on systematic reviews and payment or honoraria for lectures and attending IMMPACT meetings from ACTTION. K. V. Patel reports grants and contracts from the U.S. Centers for Disease Control and Prevention and the National Institutes of Health and consultancy work for GlaxoSmithKline LLC. Prof Rice reports support for the present manuscript from IMMPACT; grants and studentships from UKRI (Medical Research Council and BBSRC), Versus Arthritis, Royal British Legion, European Commission, UK Ministry of Defence, Dr Jennie Gwynn Bequests, Alan and Sheila Diamond Trust, the British Pain Society, and the Royal Society of Medicine; consultancy and advisory board work for Imperial College Consultants, which, in the past 36 months, has included remunerated work for Confo, Vertex, PharmaNova, Lateral, Novartis, Mundipharma, Orion, Shanghai SIMR Biotech, Asahi Kasei and Toray; and lecture honoraria from MD Anderson Cancer Center, Royal Marsden Hospital, and UCSF. A. S. C. Rice is named as an inventor on patents: A. S. C. Rice, S. Vandevoorde, and D. M. Lambert Methods using N-(2-propenyl)hexadecanamide and related amides to relieve pain. WO 2005/079771 and K. Okuse et al. Methods of treating pain by inhibition of vgf activity EP13702262.0/WO2013 110945; a role as Chair of the Trial Steering Committee (TSC) for the OPTION-DM trial, National Institute for Health Research (NIHR); a role as councilor for IASP; he also was the owner of share options in Spinifex Pharmaceuticals from which personal benefit accrued on the acquisition of Spinifex by Novartis in July 2015 (the final payment was made in 2019); and other interests are in the British National Formulary, Joint Committee on Vaccine and Immunisation-varicella subcommittee, Medicines and Healthcare products Regulatory Agency (MHRA), Commission on Human Medicines- Neurology, Pain, & Psychiatry Expert Advisory Group, Nonfreezing Cold Injury Independent Senior Advisory Committee (NISAC), and Royal College of Anaesthetists—Heritage and Archives Committee. M. C. Rowbotham reports consulting fees from SiteOne Therapeutics, GenEdit, and Sustained Therapeutics; payments for expert testimony from Haapala, Thompson & Abern (law firm for clinical payment to himself as medical-legal expert witness), payments from Helixmith Co, Ltd for work on a data monitoring or advisory board, and unpaid work as Treasurer of the International Association for the Study of Pain from 2020 to 2024; and holds stock options from SiteOne and CODA Biotherapeutics. F. Sandbrink reports a role as the National Program Executive Director for Pain Management, Opioid Safety, and Prescription Drug Monitoring Program, Veterans Health Administration. K. Schmader reports a grant by GSK for vaccine research, paid to his institution. D. J. Steiner reports being full-time employee of Eli Lilly and Company (Pain & Neurodegeneration). L. Simon reports consulting fees from AstraZeneca, Pfizer, Rigel, Eupraxia, Biosplice, EMDSerono, Horizon, Direct, Lilly, Kiniska, Protalix, Chemomab, TLC, SpineThera, Kyoto, PPD, Galvani, Urica, Transcode, Boehringer Ingelheim, Bristol Myers Squibb, Priovant, Roivant, Ampio, Aura, Aurinia, GSK, Xalud, Neumentum, Neema, Amzell, Applied Bio, Aptinyx, Bexson, Bone Med, Bone Therapeutics, Cancer Prevention, Cerebral Therapeutics, ChemoCentryx, Diffusion Bio, Elorac, Enalare, Foundry Therapeutics, Galapagos. Histogen, Gilead, Idera, Intravital, InGel, Kiel Labs, Mesoblast, Mpathix, Minerva, Regenosine, Samus, Sana, StageBio, Theraly, Unity, and Viridian. D. C. Turk reports royalties and licenses from Wolters Kluwer (Editor-n-Chief, Clinical Journal of Pain) and the American Psychological Association (Book Author); consulting fees from GSK/Novartis; and a role as Associate Director of Analgesic, Anesthetic, and Addiction Clinical Trials, Innovations, Opportunities and Networks (ACTTION). C. Veasley reports no conflicts of interest. J. Vollert reports consulting fees from Vertex Pharmaceuticals, Embody Orthopaedic, and Casquar. Finally, A. D. Wasan reports no conflicts of interest.
